# Why sleep assessment belongs in the memory clinic

**DOI:** 10.1002/alz.71880

**Published:** 2026-10-25

**Authors:** Anastasia Suraev, Mariana Fernandes, Andrew Lim, Fang Han, Sharon L. Naismith, Claudio Liguori

**Affiliations:** ^1^ Healthy Brain Ageing Program, Brain and Mind Centre Faculty of Science University of Sydney Camperdown New South Wales Australia; ^2^ Charles Perkins Centre University of Sydney Camperdown New South Wales Australia; ^3^ Department of Systems Medicine University of Rome Tor Vergata Rome Italy; ^4^ Neurology Unit University Hospital of Rome Tor Vergata Rome Italy; ^5^ Division of Neurology Department of Medicine, Hurvitz Brain Sciences Program, Sunnybrook Health Sciences Centre University of Toronto Toronto Ontario Canada; ^6^ The Division of Sleep Medicine Peking University People's Hospital Beijing China; ^7^ PKU‐IDG/McGovern Institute for Brain Research Beijing China; ^8^ IRCCS Neuromed Pozzilli (IS) Italy

**Keywords:** cognitive aging, mild cognitive impairment, risk reduction, sleep screening, stepped care

## Abstract

Sleep disturbances are highly prevalent in older adults and are particularly common among individuals attending memory clinics for assessment of cognitive impairment. Emerging evidence links sleep disorders, including insomnia, obstructive sleep apnea, circadian rhythm disruption, and rapid eye movement sleep behavior disorder, to cognitive decline, neurodegenerative pathology, and reduced quality of life for patients and caregivers. Despite their high prevalence and clinical relevance, sleep disturbances are not routinely assessed within many memory clinic and neurologic, psychiatric, and geriatric settings, and approaches to screening, diagnosis, and management vary widely across health‐care systems. This Perspective article examines current approaches to sleep assessment and management within memory clinic and specialized settings across four international contexts. We highlight the absence of systematic approaches to identifying and managing sleep disturbances in this population and propose the need for a pragmatic stepped‐care framework to improve detection, guide referral pathways, and support more consistent and personalized care in memory clinic settings.

## Highlights

- Sleep disturbances are highly prevalent in memory clinic populations.
- Sleep assessment is inconsistently integrated into memory clinic care.
- Current guidelines provide limited practical guidance for sleep assessment.
- Access to objective testing and specialist sleep services varies widely.
- Stepped‐care frameworks may facilitate routine sleep assessment.

## 1 INTRODUCTION

Sleep is a fundamental biological process supporting memory consolidation, synaptic plasticity, and overall brain health. Recent studies demonstrate that learning and memory depend on the integrity and quantity of deep sleep,^1^ that even short‐term sleep disruption can impair learning and recall,^2^ and that sleep disorders are associated with increased risk of cognitive impairment and vulnerability to all‐cause dementia.^3^ Sleep disturbances are highly prevalent in older adults, affecting up to 40% to 50% of community‐dwelling individuals,^4^ with substantially higher rates among those with cognitive impairment.^5^ They impair quality of life^6^ and place a burden on caregivers, and are often managed with sedative medications such as benzodiazepines, sedative antidepressants, and antipsychotics despite well‐recognized risks in older populations.^7^ Despite their high prevalence and growing evidence linking sleep disturbances to neurodegeneration and dementia risk, systematic approaches for identifying and managing sleep disorders in memory clinic populations remain poorly defined.

Memory clinics play a central role in the early identification and diagnosis of cognitive impairment and in initiating strategies aimed at risk reduction and symptom management. Within this setting, sleep disturbances are increasingly recognized as clinically important and have been implicated in both cognitive symptoms and underlying disease processes.^8^, ^9^, ^10^ Recent evidence indicates that comorbid insomnia and obstructive sleep apnea (COMISA) is associated with greater cognitive and behavioral impairment.^11^ In addition, increasing attention has been directed toward sleep–wake rhythms, as circadian disruption may contribute to reduced daytime functioning and poorer clinical outcomes.^12^

Obstructive sleep apnea (OSA) has been associated with poorer global cognition and episodic memory,^8^, ^13^, ^14^ while abnormal sleep duration (short and long sleep) has been linked to multi‐domain cognitive deficits and an increased likelihood of amnestic mild cognitive impairment (MCI).^14^ Untreated OSA has been linked to structural compromise in temporal brain regions involved in learning and memory,^13^ with rapid eye movement (REM)‐related hypoxemia associated with hippocampal CA1 vulnerability.^8^ These findings support routine screening for OSA and nocturnal hypoxemia in memory clinic settings, particularly given emerging evidence that treatment may benefit cognitive outcomes in MCI.^15^ Emerging work in Australian cohorts also suggests that, in addition to hypoxemia and sleep fragmentation, sleep *micro*architecture—including spindle activity—may help define distinct clinical phenotypes.^16^ Advanced circadian timing and changes to rest–activity rhythms may also emerge early in the course of cognitive decline, and may be linked with depressive symptoms^17^ and structural brain changes.^18^ Sleep symptoms are closely intertwined with depressive and neuropsychiatric symptoms, with depressive symptoms being intrinsically linked to, and potentially exacerbated by, sleep disturbance in older adults attending memory clinics.^19^ From a neurobiological perspective, sleep disturbances have been associated with structural^13^ and functional^20^ brain changes, increased amyloid^21^ and tau deposition,^22^ as well as alterations within the orexin system^23^ and neuromodulatory systems.^24^ These findings support the clinical importance and potential mechanistic role of sleep disturbances in cognitive aging. Despite the growing clinical relevance of sleep disturbances and their potential involvement in pathways underlying cognitive aging and neurodegeneration, there remains substantial variation in whether and how sleep disturbances are screened, investigated, and managed in memory clinic populations.

In this Perspective article, we first examine the prevalence and clinical burden of sleep disturbances, current policy and guideline frameworks, screening and diagnostic practices, and access to treatment and specialist services across four international health‐care systems. These settings are presented as illustrative examples of how health‐care organization, reimbursement, and clinical culture influence sleep assessment and management in memory clinic populations. We then propose a pragmatic, evidence‐informed framework for sleep assessment in memory clinic settings that outlines general principles of care and can be adapted to local resources, clinical pathways, health‐care systems, and country‐specific treatment availability.

## 2 INTERNATIONAL APPROACHES TO SLEEP SCREENING AND MANAGEMENT IN MEMORY CLINIC AND GERIATRIC SETTINGS

### 2.1 Europe

#### 2.1.1 Prevalence and clinical burden

Europe currently has the oldest population in the world,^25^ and Italy is among the most aged countries, with ~25% of its population aged ≥ 65. Approximately two million people in Italy live with MCI or dementia, with a further four million individuals involved in their care.^26^ Prevalence estimates for sleep disorders vary across assessment methods, but 25% to 90% of people with dementia are estimated to experience at least one sleep disorder. The most frequent are OSA, insomnia, and restless legs syndrome (RLS), whereas excessive daytime sleepiness represents a challenging symptom frequently related to OSA or circadian sleep–wake rhythm disorders. REM sleep behavior disorder (RBD) is less prevalent in all‐cause dementia but has a higher prevalence in synucleinopathies.^27^

Evidence from European cohorts further highlights the burden of sleep disorders in this population. In a Dutch memory clinic cohort, 17.3% of patients had a prior or newly diagnosed OSA, with prevalence highest in those with subjective cognitive complaints (26.7%), MCI (13.8%), and dementia (8%); however, OSA was objectively assessed only when clinically suspected, suggesting that the true prevalence may be underestimated.^28^ Similarly, in a multicenter Italian study of 431 individuals, sleep disturbances were identified in 65.2% of those with MCI and in 66% to 90% of those with dementia, encompassing insomnia, circadian rhythm sleep–wake disorders, sleep‐disordered breathing, RBD, and RLS.^5^ These disturbances impose a substantial clinical burden, significantly affecting the quality of life of both patients and caregivers. In care home residents, clinically significant sleep disturbances are linked to higher distress among residents and staff, as well as greater use of psychotropic medications.^29^

#### 2.1.2 Current policy and guideline frameworks

In 2014, the sleep study group of the Italian Association for Dementia, within the Italian Neurological Society, published the only Italian guidelines on the clinical assessment and management of sleep disorders in individuals with MCI and dementia.^30^ Unfortunately, this initiative has not been followed by others, leaving a significant gap between clinical needs and the applicability of screening in populations at risk. The 2024 dementia and MCI guidelines from the Italian Ministry of Health and the National Institute of Health recommend a personalized multicomponent approach to sleep management, including sleep hygiene education, daylight exposure, and exercise, while underlining the diagnostic role of RBD in Lewy body dementia. At the European level, the “Prioritization Process for European Academy of Neurology Clinical Practice Guidelines”^31^ identified “sleep and cognitive decline” as a topic requiring development. However, in current position statements, sleep hygiene is included only as general lifestyle advice within broader brain health recommendations, with limited specific guidance on clinical assessment or management.^32^

#### 2.1.3 Screening and diagnostic practices

In clinical practice, instrumental investigations are often not readily available or reimbursed in dementia centers; therefore, clinical history and structured sleep interviews remain fundamental. However, for disorders like OSA and RBD, objective sleep testing is necessary for diagnosis. While some patients with cognitive impairment may have difficulty tolerating or completing complex assessments, many can successfully undergo attended overnight polysomnography (PSG) conducted in sleep laboratories with video monitoring (Type I/video‐PSG), unattended full PSG performed at home (Type II PSG), or portable sleep studies used primarily to assess sleep‐disordered breathing (Type III sleep study). Simplified tools like actigraphy have been increasingly used, particularly in patients who have difficulty tolerating or completing more complex assessments. Actigraphy has an established role in assessing insomnia and circadian rhythm sleep–wake disorders and may provide an intermediate step between clinical assessment and PSG. New contact and non‐contact sleep monitoring technologies are rapidly developing but still require further validation, although they represent promising tools for the future research agenda.

#### 2.1.4 Access to treatment and specialist services

Assessment and management of sleep and circadian disturbances in dementia patients remain challenging and may appear time consuming; however, these processes are essential to avoid inappropriate pharmacological treatment but also to identify potential new therapeutic strategies. Across Europe, this effort aligns with the European Sleep Research Society (ESRS) guidelines, yet its clinical implementation remains highly heterogeneous due to disparate reimbursement policies and varying levels of specialized training across EU member states. Among the national health‐care systems in Europe, this is further complicated by severe structural gaps, including a significant shortage of accredited sleep medicine specialists and dedicated regional centers. Additionally, appropriate identification and management of sleep disorders may improve sleep‐related outcomes for both patients and caregivers while aiding prevention efforts. Achieving this requires a coordinated European effort to integrate sleep medicine into the broader European strategies for neurodegenerative diseases and brain health. In Italy, this translates into an urgent need to optimize the clinical network by bridging the gap between Centers for Cognitive Disorders and Dementia (CCDDs) and neurological sleep clinics. Integrating standardized sleep and clinimetric evaluations directly into the territorial neurological network is crucial to overcome current fragmentation, reduce long waiting lists for advanced diagnostics like Type I and II polysomnography, and prevent the inappropriate or prolonged use of drugs for sleep disorders, which remains a widespread concern at both national and European levels.

### 2.2 Australia

#### 2.2.1 Prevalence and clinical burden

Australia faces a rapidly aging population, with dementia now the leading cause of death.^33^ Sleep disturbances are highly prevalent among older Australians with cognitive concerns. In a cohort of > 1200 adults aged ≥ 50 years attending a specialist brain health and memory clinic, more than half reported poor sleep quality, 12% met criteria for clinically significant insomnia, and 14% endorsed symptoms consistent with RBD. Although < 15% had a prior diagnosis of OSA, among those who underwent PSG, 74% met OSA criteria, with 40% in the moderate–severe range.^14^

#### 2.2.2 Current policy and guideline frameworks

Australia's Clinical Practice Guidelines and Principles of Care for People with Dementia provide limited sleep‐specific recommendations. By contrast, the 2024 Australian Dementia Network Memory Clinic Guidelines recommend routine sleep screening in memory clinics;^34^ however, they do not provide detailed guidance regarding recommended screening instruments, assessment pathways, referral processes, or management approaches. Furthermore, available data on sleep screening practices in Australian memory clinics remain scarce, and evidence prior to the Guidelines suggests low uptake of structured sleep screening tools.^35^

#### 2.2.3 Screening and diagnostic practices

A survey of Australian memory clinic clinicians found that only 32% reported using the Epworth Sleepiness Scale and 36% the Pittsburgh Sleep Quality Index (PSQI) at least “sometimes,” while routine use (“more than half of the time”) was reported by just 4% and 6% of clinicians, respectively.^35^ As a result, assessment often relies on clinical judgment. Although some patients present with pre‐existing OSA diagnoses,^36^ self‐report questionnaires (e.g., STOP‐BANG, Multivariate Apnea Index) may under‐detect OSA in those with cognitive impairment,^36^, ^37^ owing to reduced reliability of symptom reporting, impaired recall, diminished symptom awareness, and the frequent need for caregiver‐informed history. Screening is further complicated by the need to assess multiple sleep domains using separate questionnaires. To address this challenge, new, multidomain screening tools (e.g., the CogSleep Screener) have recently been developed for use in memory clinic settings.^38^

When screening suggests a sleep disorder for which objective confirmation is clinically indicated, particularly OSA, RBD, or other complex sleep disorders, further objective assessment is required. Overnight pulse oximetry shows acceptable diagnostic performance compared to PSG and may provide a feasible screening approach to help exclude moderate‐to‐severe OSA in memory clinic settings.^36^ While PSG remains the reference standard for comprehensive assessment and is required for confirmation of disorders such as RBD, this usually involves referral to external sleep laboratories, long wait times, and out‐of‐pocket costs. Home‐based studies may be a pragmatic alternative but still require specialist sleep expertise.

#### 2.2.4 Access to treatment and specialist services

The provision of interventions targeting sleep–wake disturbances in memory clinic populations remains limited. Only 36.4% of memory clinics report offering treatments addressing sleep–wake functioning,^39^ despite these now being recommended in national memory clinic guidelines and consensus statements.^34^, ^40^ Data describing the interventions routinely delivered in these settings are sparse. For patients with OSA, treatment options such as continuous positive airway pressure (CPAP) therapy and mandibular advancement devices may be recommended. Although some individuals with cognitive impairment may experience difficulties tolerating CPAP, as may cognitively unimpaired patients, many can tolerate and benefit from treatment, and cognitive impairment or dementia alone should not preclude its prescription. The uptake and implementation of these interventions among people with MCI or dementia, including within memory clinic settings, remain poorly characterized. In line with this, recent literature emphasizes the need for personalized approaches to optimize CPAP adherence in individuals with dementia and cognitive decline, including strategies to improve tolerability and address individual barriers to treatment.

There is increasing recognition of the need to minimize benzodiazepine and sedative medications (antidepressants and antipsychotics) use in older adults due to concerns regarding falls, cognitive impairment, and dependence,^41^ with the notable exception of clonazepam as the appropriate treatment for RBD. For insomnia, the non‐benzodiazepine hypnotics (“Z‐drugs”) zopiclone and zolpidem are approved by the Therapeutic Goods Administration (TGA) in Australia but are recommended only for short‐term use and are not routinely subsidized under the Pharmaceutical Benefits Scheme (PBS). Prolonged‐release melatonin is also prescription only and not PBS subsidized, although pharmacists may supply it for short‐term treatment in adults aged > 55 years. The dual orexin receptor antagonists (DORAs), suvorexant and lemborexant, are approved for insomnia characterized by difficulties with sleep initiation or maintenance but are likewise not PBS subsidized. Consequently, access to pharmacological sleep treatments often involves substantial out‐of‐pocket costs, contributing to variability in prescribing practices and patient access. Non‐pharmacological approaches, particularly cognitive behavioral therapy for insomnia (CBT‐I), are considered effective for treating insomnia in older adults, including those with MCI;^42^ however, anecdotally, their availability for use in memory clinics is rare. Australia's mixed funding model, combining Commonwealth‐supported private services (partially subsidized through Medicare with additional out‐of‐pocket costs) and state‐ and territory‐funded public services, may further contribute to variation in care delivery.

### 2.3 China

#### 2.3.1 Prevalence and clinical burden

Epidemiological data on sleep disorders in people with dementia remain limited in China; however, available evidence suggests a substantial burden, with one study reporting sleep disorders in 55.9% of individuals with Alzheimer's disease (AD).^43^ Among community‐dwelling older adults, sleep disturbances are more common in those with MCI than cognitively healthy peers and are associated with depressive symptoms, multimorbidity, and physical inactivity, supporting routine assessment of sleep disturbances as part of comprehensive cognitive care.^44^ However, evidence describing sleep assessment and management practices within Chinese memory clinics remains scarce.

#### 2.3.2 Current policy and guideline frameworks

In China, the assessment and management of sleep disorders in people with cognitive decline are increasingly recognized as integral to dementia care, guided by recent expert consensuses and guidelines. Nationally, the integration of sleep assessment into dementia care guidelines remains limited. While two scholars from mainland China proposed a diagnostic and treatment framework for sleep–wake disorders in patients with cognitive impairment in 2021, no widely adopted national or internationally recognized guideline currently exists addressing sleep disorders in this population. Similarly, the 2018 “Chinese Guidelines for the Establishment of Memory Clinics” acknowledge the diagnostic role of PSG in diagnosing certain sleep disorders but provide no specific recommendations regarding the routine assessment of sleep disturbances, the use of screening tools, or structured diagnostic pathways within memory clinic settings. National initiatives, including the Healthy China Action (2019–2030) and the National Action Plan for Addressing Alzheimer's Disease in Old Age (2024–2030), provide a policy framework that could facilitate greater integration of sleep health into dementia care.

#### 2.3.3 Screening and diagnostic practices

Sleep assessment for older adults with cognitive impairment is most commonly conducted in community health‐care settings, nursing homes, or mental health centers rather than within memory clinics. Although memory clinics in China are expanding and many tertiary centers have access to PSG, routine sleep screening has not yet been integrated into standard memory clinic practice. Assessment typically begins with a detailed clinical history focusing on sleep–wake patterns, sleep quality, nocturnal behaviors, and daytime functioning. For individuals with moderate‐to‐severe dementia, clinicians often rely heavily on caregiver reports to obtain an accurate history of nocturnal symptoms and behavioral disturbances. The PSQI is the most commonly recommended instrument for evaluating overall sleep quality, while disorder‐specific tools such as the REM Sleep Behavior Disorder Screening Questionnaire (RBDSQ) and the Berlin Questionnaire for OSA may be used when clinically appropriate. When available and clinically indicated, PSG remains the gold standard for diagnosing disorders such as OSA and RBD, with actigraphy increasingly used as an adjunctive objective assessment tool.

#### 2.3.4 Access to treatment and specialist services

Several barriers continue to limit effective identification and management of sleep disorders in people with cognitive impairment. Sleep disturbances are not routinely incorporated as mandatory screening items in most memory clinics or geriatric departments, contributing to low recognition of conditions such as OSA, RBD, and circadian rhythm disturbances, which may be misattributed to “normal aging.” Treatment remains inconsistent, with limited implementation of non‐pharmacological interventions such as CBT‐I, light therapy, and circadian‐based interventions, while sedative–hypnotic medications continue to be commonly prescribed despite their potential risks in older adults. Access to specialist sleep services also varies considerably, with therapies including CPAP, melatonin, and light therapy devices often requiring out‐of‐pocket payment and sleep medicine expertise remaining scarce in many primary care and rural settings. Multidisciplinary collaboration between memory clinics, geriatrics, sleep medicine, and psychiatry remains limited, and structured pathways for long‐term sleep management are largely absent.

### 2.4 Canada

#### 2.4.1 Prevalence and clinical burden

Longitudinal evidence from the Canadian Longitudinal Study on Aging indicates that insomnia is associated with increased odds of subjective memory decline, and that worsening sleep over time is linked to a greater likelihood of memory decline, supporting sleep as a potentially modifiable risk factor for cognitive decline.^45^ More recently, sleep‐onset insomnia has also been associated with cognitive, motor, and autonomic features of prodromal neurodegeneration, suggesting that insomnia subtypes may provide additional insight into neurodegenerative risk.^46^ In Canadian long‐term care facilities, where cognitive impairment and dementia are highly prevalent, sleep disturbances affect ≈ 22% of residents, although prevalence varies substantially across facilities and commonly co‐occur with pain, depression, daytime sleepiness, fatigue, and psychotropic medication use, contributing to greater clinical complexity and care needs.^47^

#### 2.4.2 Current policy and guideline frameworks

In Canada, clinical practice regarding sleep disturbances and dementia is shaped in part by clinical guideline recommendations and in part by the public health‐care coverage landscape. The most recent “Canadian Consensus Conference on the Diagnosis and Treatment of Dementia” guidelines, published in 2020, addressed sleep disturbances as both markers^48^ and potential risk factors^49^ for dementia and dementia‐associated brain changes. The guidelines identify a range of sleep abnormalities associated with increased dementia risk or underlying neurodegenerative pathology, including RBD as a marker of synucleinopathies, excessive sleep duration, daytime sleepiness, napping, insomnia, sleep fragmentation, decreased non‐REM slow wave activity, altered sleep spindles, and decreased REM sleep. From a dementia risk reduction perspective, the guidelines noted the lack of randomized controlled trial evidence demonstrating that treatment of sleep disturbances reduces dementia risk. Weak recommendations were made to treat adults with OSA using CPAP, based primarily on improvements in daytime sleepiness and observational evidence suggesting potential effects on dementia‐related biomarkers, and to avoid severe sleep deprivation (< 5 hours/night). However, there was insufficient evidence to recommend treatment of insomnia, long sleep duration, daytime napping, sleep fragmentation, circadian irregularity, or abnormal circadian phase with the specific goal of improving cognition or reducing dementia risk, and substantial causal ambiguity was noted.

#### 2.4.3 Screening and diagnostic practices

Published data describing routine sleep screening practices within Canadian memory clinics are limited. Clinicians are typically advised to obtain a careful sleep history in adults with, or at risk for, cognitive impairment and dementia, and to refer patients to specialized sleep clinics for objective assessment with PSG or other modalities when clinical history suggests a sleep disorder. However, existing guidelines do not recommend routine screening for sleep disturbances in the absence of suggestive symptoms.

#### 2.4.4 Access to treatment and specialist services

Health‐care coverage is an important determinant of access to sleep‐related services in Canada. As health‐care delivery falls under provincial jurisdiction, coverage and access to sleep‐related services vary across provinces. In‐laboratory PSG is publicly funded across provinces, although access varies substantially depending on local service capacity and wait times. Home sleep apnea testing (HSAT) is covered in some provinces but not others, and coverage for CPAP therapy similarly varies. Public coverage for psychologist‐delivered CBT‐I is also inconsistent. As a result, across Canadian jurisdictions there is likely under‐diagnosis and under‐treatment of OSA, RLS, and other sleep disorders, as well as under‐use of CBT‐I for insomnia. Overall, clinical management of sleep disturbances in relation to dementia in Canada reflects both the conservative tone of current guideline recommendations and the practical constraints imposed by variable health‐care coverage and service availability.

## 3 COMMON CHALLENGES IN SLEEP ASSESSMENT ACROSS MEMORY CLINICS

Across the four health‐care systems examined, a common disconnect exists between the growing recognition of sleep disturbances as an important component of cognitive health and their limited integration into routine memory clinic care.

First, routine sleep assessment has not been systematically embedded within memory clinic pathways. Although sleep disturbances are highly prevalent among people with cognitive impairment, screening is often symptom driven rather than routinely incorporated into cognitive assessments. This contributes to variable recognition of sleep disorders and missed opportunities to identify potentially modifiable contributors to cognitive symptoms.

Second, diagnostic pathways are highly dependent on local infrastructure, workforce capacity, and health‐care funding models. Access to objective assessments such as PSG, specialist sleep services, and validated diagnostic pathways varies considerably between countries and health‐care settings, with clinical practice frequently shaped by local expertise and reimbursement structures rather than consensus guidance. Similar barriers extend to management, including limited access to specialist sleep services, long wait times and shortages of trained providers for non‐pharmacological treatments such as CBT‐I, and financial and system‐level constraints affecting access to diagnostic testing (e.g., PSG), which can delay initiation of treatments such as CPAP or mandibular advancement devices. In addition, out‐of‐pocket costs may limit access to both device‐based therapies and pharmacological treatments. These system‐level factors contribute to substantial variability in the diagnosis and management of sleep disorders across memory clinics.

Third, assessment of sleep disturbances in people with cognitive impairment presents unique clinical challenges. Reliance on self‐reported sleep symptoms may be unreliable because of impaired insight, recall, and symptom awareness, while existing screening instruments have important limitations in cognitively impaired populations. Consequently, caregiver reports, collateral history, and objective assessment, where feasible, are often essential to improve diagnostic accuracy.

These challenges highlight the need for pragmatic clinical practice frameworks capable of integrating sleep assessment and management into routine memory clinic pathways. A structured approach could standardize when and how sleep is assessed, guide escalation to objective investigations or specialist referral according to local resources, and support assessment strategies appropriate for people with cognitive impairment. In doing so, it could facilitate the integration of sleep health into comprehensive dementia care.

## 4 THE CASE FOR STEPPED‐CARE APPROACHES TO SLEEP ASSESSMENT AND MANAGEMENT OF SLEEP DISTURBANCES IN MEMORY CLINICS

Memory clinics sit at a critical juncture between early cognitive change, risk stratification, and secondary prevention. Data from the Australian Dementia Network (ADNeT) Registry Annual Report indicate that more than one third of individuals presenting to memory clinics are diagnosed with MCI, a stage at which opportunities for intervention and secondary prevention are greatest. Sleep disturbances may precede cognitive decline by many years, highlighting the potential value of incorporating routine sleep screening into memory clinic pathways. Earlier identification and management of sleep disturbances may not only improve physical health, psychological functioning, and overall quality of life but also represent a potential modifiable target within dementia risk reduction strategies. However, in the absence of clear guidance specific to cognitively impaired populations, clinical practice often defaults to general population indications for sleep assessment and treatment.

Several implementation models could support the integration of sleep assessment into memory clinic pathways, including stepped‐care frameworks, clinical decision algorithms, and integrated care pathways. Among these, stepped‐care approaches may be particularly practical in resource‐constrained settings, allowing clinicians to escalate assessment and management based on symptom severity and clinical suspicion. Importantly, the precise configuration of such pathways will depend on local infrastructure, service capacity, and access to specialist sleep services. While an overarching international consensus framework could help guide best practice in sleep assessment within memory clinics, its implementation would inevitably require adaptation to country‐specific health systems, regulatory environments, and service availability. Close collaboration between memory and sleep specialists may further support tailored approaches to the assessment and management of sleep disturbances, including consideration of sex‐specific risk profiles.^50^

## 5 A STRUCTURED FRAMEWORK FOR ASSESSMENT

A recently published framework developed within the Italian Association for the Study of Dementia (SINdem) Sleep Study Group provides a useful foundation for a stepped approach to assessing sleep and circadian disturbances in people with cognitive decline and dementia.^51^ The framework begins with low‐burden clinical assessment, including sleep history, questionnaires, and rating scales, before escalating to intermediate objective tools such as actigraphy and HSAT, and then to PSG or video‐PSG when clinically indicated. In the original framework, questionnaire selection is stratified by cognitive severity using the Clinical Dementia Rating (CDR), with the broader principle that assessment should proceed only as far as necessary to reach diagnostic sufficiency.

We adapted this framework for broader international use as a three‐tier pathway, with decision points for escalation, guidance on increasing informant input as cognitive impairment progresses, and annotations indicating where local adaptation is expected (Figure 1). The adapted framework specifies the functional domains that should be assessed at each step: sleep quality; insomnia symptoms; excessive daytime sleepiness; circadian timing and regularity; and disorder‐specific screening for OSA, RLS, and RBD. Cognitive staging may be guided by CDR where routinely used, or approximated using cognitive screening tools such as the Mini‐Mental State Examination or Montreal Cognitive Assessment when CDR assessment is not available. Patients need not progress sequentially through each tier; direct escalation to more comprehensive assessment may be appropriate when clinical features indicate a complex or high‐risk sleep disorder requiring PSG or video‐PSG.

**FIGURE 1:**
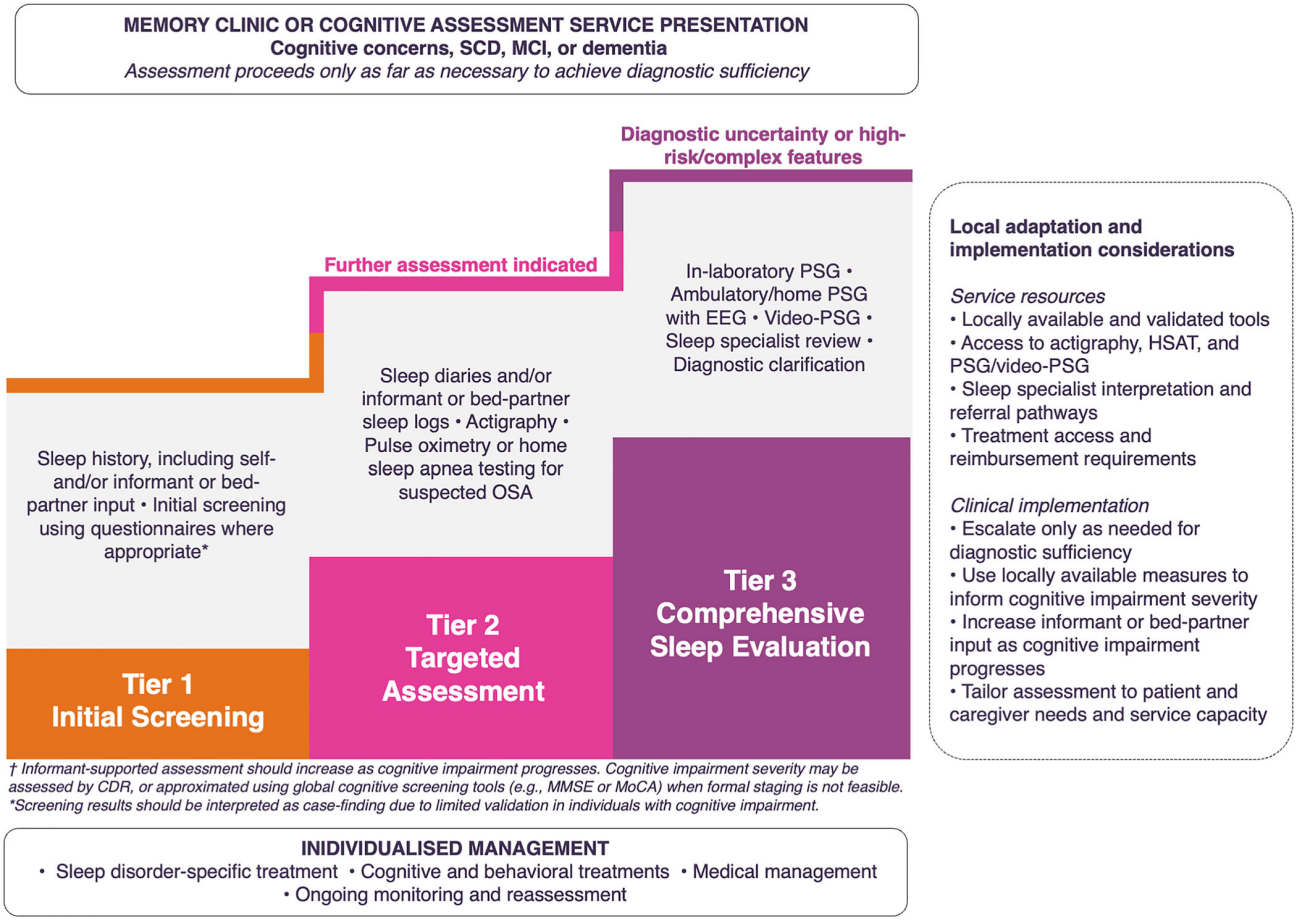
Stepped‐care framework for sleep assessment in memory clinic populations. Adapted from the Italian Association for the Study of Dementia (SINdem) Sleep Study Group, this three‐tier model progresses from initial screening through to targeted assessment and, when indicated, to comprehensive sleep evaluation, with escalation guided by clinical indication, diagnostic uncertainty, high‐risk or complex features, and diagnostic sufficiency. The framework is intended to be adapted to local resources, informant availability, and health‐care system requirements. CDR, Clinical Dementia Rating; EEG, electroencephalography; HSAT, home sleep apnea testing; MCI, mild cognitive impairment; MMSE, Mini‐Mental State Examination; MoCA, Montreal Cognitive Assessment; OSA, obstructive sleep apnea; PSG, polysomnography; RBD, rapid eye movement sleep behavior disorder; RLS, restless legs syndrome; SCD, subjective cognitive decline; video‐PSG, video‐polysomnography.

At Tier 1, initial screening should begin with a brief structured sleep history, supplemented when appropriate by informant or bed‐partner input. This should cover sleep quality, insomnia symptoms, excessive daytime sleepiness, sleep timing and regularity, snoring and witnessed apneas, dream enactment behaviors, RLS symptoms or nocturnal limb movements, and the impact of sleep disturbance on daytime functioning. The CogSleep Screener, a brief patient‐facing tool developed and validated for people with cognitive impairment, may provide a practical starting point for identifying common sleep disturbance domains.^38^ The CogSleep Semi‐Structured Interview can further guide clinicians in systematically exploring these domains, with informant input when self‐report is limited.^52^ Representative assessment tools across these domains, including self‐report questionnaires and objective assessment methods, are summarized in Table S1 in supporting information. Importantly, few self‐report questionnaires have been specifically adapted or psychometrically validated for people with cognitive impairment, and their performance may differ from that observed in cognitively healthy adults. For example, the diagnostic accuracy of commonly used OSA screening tools such as STOP‐BANG appears to decline with increasing cognitive impairment,^36^ while questionnaire‐based screening for RBD may substantially overestimate true prevalence because it screens for possible dream enactment behavior but cannot confirm the PSG hallmark of RBD, namely REM sleep without atonia, and may be confounded by conditions such as OSA and non‐REM parasomnias.^53^ Screening results should therefore be interpreted as case finding rather than diagnostic, considered within the broader clinical context, and supplemented by objective assessment when appropriate. This limitation is consistent with international consensus recommendations calling for existing sleep questionnaires to be re‐validated, scoring approaches to be adapted, and new tools to be developed for older adults with cognitive impairment.^54^ When self‐report reliability is reduced, informant‐ or bed partner–assisted assessment should be prioritized, particularly for symptoms patients may under‐recognize, including snoring, witnessed apneas, dream enactment behavior, nocturnal movements, restless legs symptoms, and daytime sleepiness.

At Tier 2, individuals with clinically relevant symptoms, persistent diagnostic uncertainty, or high‐risk features may proceed to targeted disorder‐specific assessment and intermediate objective assessment methods (see also Table S1). These may include actigraphy, sleep diaries or informant sleep logs, overnight pulse oximetry, and HSAT. Actigraphy can provide multi‐night assessment of sleep timing, sleep continuity, daytime rest or napping, and circadian rest–activity rhythms. Overnight pulse oximetry or HSAT may support assessment of suspected OSA when access to PSG is delayed or limited by local service availability, including triage of individuals who may require further evaluation. Emerging wearable, nearable, airable, and contactless sleep‐monitoring technologies may also support low‐burden assessment of sleep–wake patterns in real‐world settings. These include body‐worn devices such as wrist‐worn or ring‐based trackers, and non‐contact systems such as under‐mattress, bedside, or room‐based sensors. Research‐ or clinical‐grade systems should be distinguished from consumer‐grade devices, as validation, regulatory oversight, access to raw data, and algorithm transparency vary substantially. Recent PSG‐comparison studies and reviews of wearable, nearable, airable, and contactless sleep technologies suggest potential utility for longitudinal monitoring, while also highlighting variable device performance across outcomes and device types, particularly for wake detection and sleep‐stage classification.^55^, ^56^, ^57^, ^58^ However, these tools remain heterogeneous, often rely on proprietary algorithms, and require further validation in older adults and cognitively impaired populations before they can be used as stand‐alone diagnostic methods.^54^

At Tier 3, referral for in‐laboratory PSG, ambulatory/home PSG with electroencephalography, or video‐PSG is indicated when diagnostic uncertainty remains after Tier 1 and Tier 2 assessment, when symptoms are complex or high risk, or when objective confirmation would alter management. When PSG access is limited, Tier 1 and Tier 2 methods may support triage and interim assessment, but targeted sleep‐service referral should be prioritized for patients with persistent symptoms, inconclusive findings, high‐risk features, or suspected disorders requiring PSG or video‐PSG confirmation. Cognitive impairment alone should not be considered a reason to withhold referral for PSG, as many people with MCI or dementia can complete testing successfully, particularly when appropriate caregiver and laboratory support is provided. PSG is particularly important for suspected central sleep apnea, periodic limb movements, complex or comorbid sleep disorders, or inconclusive HSAT findings, while video‐PSG is important when RBD, complex parasomnias, nocturnal seizures, or coexisting OSA and parasomnia‐like behaviors are suspected.

Within this framework, management should be individualized according to the sleep disturbance identified, level of cognitive impairment, patient and caregiver goals, and local health‐system capacity, including referral pathways and access to behavioral, medical, and sleep‐specialist care. Table 1 summarizes practical management strategies for common sleep disturbances encountered in memory clinic populations.

**TABLE 1:** Clinical approaches to the management of sleep disorders in memory and geriatric clinic settings.

Data and guidelines available	No widely adopted guidelines for routine sleep assessment in memory clinic settings. The European Academy of Neurology has identified “sleep and cognitive decline” as a priority area for guideline development,^31^ but current position statements lack specific recommendations.^32^ Some national recommendations exist, including those published in Italy for assessment and management of sleep disorders in individuals with MCI and dementia.^30^ Australian memory clinic guidelines recommend general assessment of sleep and pathways for sleep‐wake interventions.^34^, ^40^ Canadian dementia guidelines recommend obtaining a sleep history, referral for suspected sleep disorders, and CPAP for OSA, but do not support routine screening or treatment of sleep disturbances to prevent cognitive decline.^48^, ^49^ China recognizes the diagnostic role of PSG but does not specifically recommend routine assessment, screening tools, or diagnostic pathways.
**Sleep disorders**	**Recommended clinical approach**
Insomnia and poor sleep quality	Cognitive Behavioral Therapy for Insomnia (CBT‐I) recommended as first‐line treatment of insomnia disorder, which has preliminary evidence supporting feasibility in MCI populations.^42^ However, access to CBT‐I is extremely limited in most centers: some free or low‐cost digital CBT‐I programs exist in select regions (e.g., This Way Up, Sleepio). Exercise interventions may be helpful.^59^ *Pharmacological options*: melatonergic agents (e.g., prolonged‐release melatonin or melatonin receptor agonists), dual orexin receptor antagonists (e.g., suvorexant and lemborexant; daridorexant is approved in Europe, Canada, and China but not currently in Australia), Z‐drugs (e.g., zolpidem, zopiclone for short‐term use, eszopiclone for use up to 6 months), or selected sedating antidepressants only where comorbid depression is present—although it does not represent the first‐line treatment for depression. Benzodiazepines and Z‐drugs are generally *not* recommended in older adults due to increased risks of falls, cognitive impairment, and dependence, although they may be prescribed in selected cases following an individualised assessment of benefits and risks.
RBD symptoms	Safety measures and environmental modifications (e.g., bedroom adaptations and injury prevention strategies). *Pharmacological treatment (if clinically indicated)*: melatonin or clonazepam.
Restless legs syndrome	For mild cases: regular physical activity, avoidance of exacerbating factors (e.g., medication triggers), and identification and treatment of iron deficiency. *Pharmacological treatment*: First‐line options include supplemental iron (oral or intravenous) and α2‐δ ligands (e.g., gabapentin or pregabalin). Recent American Academy of Sleep Medicine clinical practice guidelines recommended against the use of non‐ergot dopamine agonists (e.g., pramipexole) due to the risk for augmentation. Opioids may be considered in severe or refractory cases (off‐label).
OSA	CPAP therapy, weight management, positional therapy, mandibular advancement splints, and in selected cases maxillofacial surgery. *Pharmacological treatment*: Tirzepatide (dual GIP/GLP‐1 receptor agonist) is approved for moderate‐to‐severe OSA in adults with obestiy in selected jurisdictions; emerging evidence supports the potential benefit of other incretin‐based therapies.
Circadian	Light therapy (evidence remains limited^60^), structured daytime activity and light exposure, and behavioral circadian stabilization. *Pharmacological treatment*: timed melatonin administration to support circadian entrainment.

*Note*: Across the health‐care systems discussed, variation appears to reflect differences in implementation, service capacity, reimbursement, referral pathways, diagnostic access, and specialist availability, rather than in recommended clinical management.

Abbreviations: CBT‐I, cognitive behavioral therapy for insomnia; CPAP, continuous positive airway pressure; GIP, glucose‐dependent insulinotropic polypeptide; GLP‐1, glucagon‐like peptide‐1; MCI, mild cognitive impairment; OSA, obstructive sleep apnea; PSG, polysomnography; RBD, rapid eye movement (REM) sleep behavior disorder.

Strong clinical recommendations for systematic sleep assessment and management in individuals with cognitive impairment should ultimately be supported by evidence of clear clinical benefit. Several potential pathways may underlie such benefit. First, specific sleep features or combinations of sleep disturbances may serve as sensitive markers of particular dementia pathologies or clinical syndromes. Second, sleep disturbances may contribute to the development or progression of dementia‐related brain changes, raising the possibility that targeted treatment could attenuate disease risk or progression. Third, neurodegenerative processes may produce cognitive and behavioral symptoms partially through disruption of sleep‐dependent processes such as memory consolidation, meaning that treatment of sleep disturbances could mitigate downstream cognitive effects. Finally, sleep disruption as a co‐morbidity may amplify the clinical impact of dementia‐related brain pathology, with treatment potentially improving cognitive or behavioral outcomes even in the presence of established disease.

Clarifying these mechanisms will require additional studies linking sleep features to underlying neuropathology, as well as randomized controlled trials testing whether detection and treatment of specific sleep abnormalities can slow cognitive decline, modify disease‐related brain changes, or enhance resilience to neurodegeneration. Future work should incorporate a broader internationally representative working group encompassing diverse geographical regions and health‐care settings to develop globally relevant consensus recommendations for sleep assessment and management in the memory clinic setting. In the interim, a pragmatic stepped‐care framework may offer a feasible approach to improving detection and management of sleep disturbances in memory clinic populations while the evidence base regarding causal and disease‐modifying effects continues to evolve.

## CONFLICT OF INTEREST STATEMENT

A.S. reports support from Haleon Australia for attendance at an advisory meeting unrelated to the present work. A.L. reports research funding from the Canadian Institutes of Health Research (CIHR), US National Institutes of Health (NIH), and Weston Family Foundation, and advisory board participation for Takeda. S.L.N. reports research funding from the National Health Medical Research Council (NHMRC) and Medical Research Future Fund (MRFF); consulting fees from Takeda, Eli Lilly, Eisai, and Roche Diagnostics; speaker honoraria from Eli Lilly; and meeting support from Novo Nordisk. All other authors report no relevant disclosures. Author disclosures are available in the Supporting Information.

## Supporting information



Supporting Information

Supporting Information
