# Supplementary material for: Why sleep assessment belongs in the memory clinic
Source: Alzheimers Dement. 2026 Oct 25;22(10):e71880. doi: 10.1002/alz.71880 (PMC13696753; doi:10.1002/alz.71880)
Supplement: Supplementary file 2 [file ALZ-22-e71880-s001.docx]

| **Table S1.** Examples of assessment tools for common sleep disturbances in memory clinic populations | |
| --- | --- |
| **Domain** | **Examples** |
| ***Sleep history*** | |
| Semi-structured interview | CogSleep Semi-Structured Sleep-Wake Interview^1^ |
| ***Questionnaires (patient- or informant-reported)*** | |
| Multidomain brief screen | CogSleep Screener^2^* |
| Sleep quality | Pittsburgh Sleep Quality Index (PSQI)^3†^ |
|  | Patient-Reported Outcomes Measurement Information System (PROMIS) Sleep Disturbance Scale^4^ |
|  | The Sleep Disorders Inventory (SDI; informant-rated)^5^* |
| Insomnia | Insomnia Severity Index (ISI)^6^ |
| Daytime sleepiness | Epworth Sleepiness Scale (ESS)^7^ |
| Obstructive sleep apnea | STOP-Bang Questionnaire^8†^ |
|  | Berlin Questionnaire^9†^ |
| REM sleep behavior disorder | Rapid Eye Movement Sleep Behaviour Disorder Screening Questionnaire (RBDSQ)^10^ |
|  | One-Question Rapid Eye Movement Sleep Behaviour Disorder Screen (RBD1Q)^11^ |
|  | International RBD Severity Scale (IRBD-SSS) – Patient (IRBD-SSS-PT) and Bed-Partner (IRBD-SSS-BP) versions^12^ |
|  | The Mayo Sleep Questionnaire (MSQ; informant/bed partner-rated)^13*^ |
| Restless legs syndrome | International Restless Leg Syndrome Rating Scale (IRLS; symptom severity)^14^ |
| Circadian preference / timing | Morningness-Eveningness Questionnaire (MEQ)^15^ and short-form version (r-MEQ)^16^ |
| ***Objective assessment*** | |
| Multi-night sleep-wake activity | Validated wrist actigraphy or accelerometry-based sleep monitoring, e.g., Actiwatch or equivalent validated devices, with self-report sleep diary |
| Oxygen saturation and heart rate | Overnight pulse oximetry, e.g., wrist-worn/finger-probe oximetry systems such as WristOx, or equivalent validated devices |
| Suspected sleep-disordered breathing | Home sleep apnea testing (HSAT), including traditional respiratory polygraphy or peripheral arterial tonometry-based systems (e.g., WatchPAT) |
| Broader ambulatory sleep assessment | Ambulatory/home PSG with EEG, including unattended (Level 2) home PSG where available |
| Confirmatory sleep assessment | In-laboratory PSG; video-PSG |
| *Abbreviations:* AD, Alzheimer's disease; EEG, electroencephalography; MCI, mild cognitive impairment; OSA, obstructive sleep apnea; PSG, polysomnography; SCD, subjective cognitive decline.  Note: Examples are illustrative and non-exhaustive; inclusion does not imply endorsement of any instrument, device, or manufacturer. Instrument selection should reflect the clinical question, the patient’s level of cognitive impairment, the availability of a reliable informant or bed partner, and local expertise and referral pathways. Unless otherwise indicated, questionnaires are patient-completed. Most self-report questionnaires have not been specifically validated in people with cognitive impairment and should be used for case-finding rather than diagnosis, supplemented by informant or bed-partner report where appropriate and objective assessment when clinically indicated.  * Validated in populations with cognitive impairment and/or memory clinic settings. ^†^ Selected questionnaires may have reduced accuracy or interpretability in memory clinic populations, including STOP-Bang for OSA screening and discrepancies between self-reported and objectively measured sleep duration and timing in SCD/MCI,^17,18^ and the STOP-Bang and Berlin Questionnaire against PSG in mild-to-moderate AD.^19^ | |

**Supplementary References**

1.	Naismith SL, Duffy S, Gordon C. *Semi-structured sleep–wake interview for older adults*. Sydney: NHMRC Centre of Research Excellence to Optimise Sleep in Brain Ageing and Neurodegeneration (CogSleep CRE); 2022. Accessed 31 July 2026. Available from: https://siesta.sydney.edu.au/wp-content/uploads/2022/04/CogSleep_Questionnaire_DTA_Version_March_2022.pdf

2.	Kong SD, Schrire ZM, Lin PH, et al. Validating the CogSleep Screener in older adults at a memory and cognition clinic. *J Sleep Res.* 2025;34(3):e14355.

3.	Buysse DJ, Reynolds CF III, Monk TH, Berman SR, Kupfer DJ. The Pittsburgh Sleep Quality Index: a new instrument for psychiatric practice and research. *Psychiatry Res*. 1989;28(2):193-213.

4.	Buysse DJ, Yu L, Moul DE, et al. Development and validation of patient-reported outcome measures for sleep disturbance and sleep-related impairments. *Sleep*. 2010;33(6):781-792.

5.	Tractenberg RE, Singer CM, Cummings JL, Thal LJ. The Sleep Disorders Inventory: an instrument for studies of sleep disturbance in persons with Alzheimer's disease. *J Sleep Res*. 2003;12(4):331-7.

6.	Bastien CH, Vallières A, Morin CM. Validation of the Insomnia Severity Index as an outcome measure for insomnia research. *Sleep Med*. 2001;2(4):297-307.

7.	Johns MW. A new method for measuring daytime sleepiness: the Epworth sleepiness scale. *Sleep*. 1991;14(6):540-545.

8.	Chung F, Abdullah HR, Liao P. STOP-Bang questionnaire: a practical approach to screen for obstructive sleep apnea. *Chest*. 2016;149(3):631-638.

9.	Netzer NC, Stoohs RA, Netzer CM, Clark K, Strohl KP. Using the Berlin Questionnaire to identify patients at risk for the sleep apnea syndrome. *Ann Intern Med*. 1999;131(7):485-491.

10.	Stiasny‐Kolster K, Mayer G, Schäfer S, Möller JC, Heinzel‐Gutenbrunner M, Oertel WH. The REM sleep behavior disorder screening questionnaire—a new diagnostic instrument. *Mov Disord*. 2007;22(16):2386-2393.

11.	Postuma RB, Arnulf I, Hogl B, et al. A single‐question screen for rapid eye movement sleep behavior disorder: a multicenter validation study. *Mov Disord*. 2012;27(7):913-916.

12.	Fantini ML, Postuma RB, Puligheddu M, et al. Validation of the international REM sleep behavior disorder study group symptoms severity scale (IRBD-SSS): a new tool to assess RBD clinical severity. *J Neurol*. 2024;271(11):7264-7271.

13.	Boeve BF, Molano JR, Ferman TJ, et al. Validation of the Mayo Sleep Questionnaire to screen for REM sleep behavior disorder in an aging and dementia cohort. *Sleep Med*. 2011;12(5):445-453.

14.	Walters AS, LeBrocq C, Dhar A, et al. Validation of the International Restless Legs Syndrome Study Group rating scale for restless legs syndrome. *Sleep Med*. 2003;4(2):121-32.

15.	Horne JA, Ostberg O. A self-assessment questionnaire to determine morningness-eveningness in human circadian rhythms. *Int J Chronobiol.* 1976;4(2):97-110.

16.	Adan A, Almirall H. Horne & Östberg morningness-eveningness questionnaire: A reduced scale. *Pers Individ Dif.* 1991;12(3):241-253.

17.	Lam A, D’Rozario AL, Kong S, et al. Screening for obstructive sleep apnea in the memory clinic: a comparison of questionnaires, pulse oximetry, and polysomnography. *J Alzheimers Dis.* 2025;103(1):218-229.

18.	Lam A, Kong D, D’Rozario AL, et al. Sleep disturbances and disorders in the memory clinic: Self-report, actigraphy, and polysomnography. *J Alzheimers Dis.* 2025;106(1):78–93.

19.	Jorge C, Benítez I, Torres G, et al. The STOP-Bang and Berlin questionnaires to identify obstructive sleep apnoea in Alzheimer's disease patients. *Sleep Med*. 2019;57:15-20.
